# Intrauterine phenotype features of fetuses with 7q11.23 microduplication syndrome

**DOI:** 10.1186/s13023-023-02923-y

**Published:** 2023-09-27

**Authors:** Yunan Wang, Chang Liu, Rong Hu, Juan Geng, Jian Lu, Xin Zhao, Ying Xiong, Jing Wu, Aihua Yin

**Affiliations:** 1 Medical Genetic Center, Guangdong Women and Children Hospital, NO.521-523, Xingnan Road, Panyu District, Guangzhou, 511442 Guangdong People’s Republic of China; 2 Maternal and Children Metabolic-Genetic Key Laboratory, Guangdong Women and Children Hospital, Guangzhou, 510010 Guangdong People’s Republic of China; 3 UItrasonic Diagnosis Deparment, Guangdong Women and Children Hospital, Guangzhou, 510010 Guangdong People’s Republic of China

**Keywords:** dup7q11.23 syndrome, Prenatal diagnosis, Sonographic features, Chromosomal microarray

## Abstract

**Objective:**

To share our experience on prenatal diagnosis of 7q11.23 microduplication syndrome and to further delineate the fetal phenotypes of the syndrome.

**Methods:**

A retrospective study was conducted to evaluate seven cases of dup7q11.23 syndrome diagnosed prenatally by chromosomal microarray (CMA). Clinical data were reviewed, including maternal characteristics, indications for prenatal diagnosis, sonographic findings, CMA results, pregnancy outcomes and follow-ups.

**Results:**

Seven cases, including 2 pairs of MCDA twins, were prenatally identified with dup7q11.23 syndrome. The most common prenatal sonographic features were ventriculomegaly, low-lying conus medullaris, and dilated ascending aorta. All 7 fetuses presented with typical 7q11.23 duplications (1.40–1.55 Mb). Parental chromosome analysis was performed in four pairs of parents, and indicated that the duplications of Case 6 and 7 were inherited from their asymptomatic mother.

**Conclusion:**

Our case series suggest that prenatal features of dup7q11.23 cases are diversified, with ventriculomegaly and low-lying conus medullaris being the most common intrauterine phenotypes. Additionally, cleft palate, dilated ascending aorta, and renal abnormalities were also observed, and should be taken into consideration in subsequent studies.

## Introduction

Williams-Beuren syndrome (WBS) (OMIM 194050) is a common microdeletion syndrome caused by interstitial deletions of the 7q11.23 region. Most 7q11.23 microdeletions are de novo, while inherited deletions are rare [1, 2]. Prenatal phenotypic features of WBS, include intrauterine growth retardation and congenital cardiovascular abnormalities. More recently, the reciprocal microduplication of the same chromosomal region implicated in WBS has been identified in several patients who underwent chromosome microarray, because of intellectual disability (ID), mild facial dysmorphism, language impairment and congenital malformations [3–5]. Parental transmission of dup7q11.23 syndrome is comparatively common [6]. Unlike its reciprocal deletion, to the best of our knowledge, there is very little information on the intrauterine phenotypes of dup7q11.23 syndrome.

This study provides a comprehensive overview of the clinical features, intrauterine phenotypes, and molecular cytogenetic results of seven fetuses with dup7q11.23 syndrome identified by single nucleotide polymorphism array (SNP-array). The results are compared with published data of fetuses with duplication 7q11.23 syndrome to identify distinguishing and shared features.

## Material and methods

### Subject

Three singleton pregnancies and two pairs of monochorionic diamniotic (MCDA) twin pregnancies were enrolled. Each case underwent a routine ultrasound scan at a primary hospital, and be referred to our Medical Genetic Center for reassessment. Their initial invasive prenatal diagnosis indications include high risk for trisomy 21 and trisomy 7 by noninvasive prenatal testing (NIPT) (case 1) and anomalies on ultrasonography (case 2–7). After ultrasound reassessment and genetic counseling, all pregnant women received genetic diagnostic testing. The mean maternal age was 27.8 years, ranging from 23 to 31 years. Follow-up data on height, weight, facial features, physical activity, adaptive behavior, language and personal-social behavior were collected in newborns. This study has been approved by the Institutional Review Board/ Medical Ethics Committee of Guangdong Women and Children Hospital (IRB reference number: 201801073). Written informed consent was obtained from each participating family.

### Cytogenetic and molecular analyses

G-banding (320–400 bands) was performed on metaphase chromosomes of amniotic fluid cells using standard procedures [7]. Genomic DNA was extracted from fetal uncultured amniotic fluids and their parents’ peripheral blood using the Lab-Aid 820 automation system (Zee San Biotech Company, Fujian, China). SNP-array analysis was performed on a commercial CytoScan 750 K Array (Affymetrix, Santa Clara, CA) containing 750,436 25-85mer oligonucleotide probes, including 550,000 nonpolymorphic probes and 200,436 SNP probes. The labeling and hybridization of the genomic DNA was performed following the manufacturer’s protocol. Results were analyzed by Affymetrix Chromosome Analysis Suite software [8].

For twin pregnancies, short tandem repeat (STR) assay was performed to determine the zygosity, and eliminate maternal contamination. Twenty-six STR loci were compared. If all loci were identical, the two fetuses were considered monozygotic.

### Medical exome sequencing

Genomic DNA was extracted using a Qiagen DNA blood mini kit (Qiagen GmbH, Hilden, Germany). Library preparation and target enrichment were performed using a SureSelectXT Clinical Research Exome kit (Agilent Technologies, Santa Clara, CA) according to the manufacturer’s specifications. Then, Trio-Medical Exome Sequencing (Trio-MES) was performed using 2 × 150 bp in the paired end mode of the NextSeq 500 platform (Illumina, San Diego, CA) to obtain an average coverage of above 110x, with 97.6% of target bases covered at least 10x. Sequence quality analysis and filtering of mapped target sequences were performed with the ‘varbank’ exome and genome analysis pipeline v.2.1 as described previously [9]. Analysis of genetic results was based on the genomic variation database (http://dgv.tcag.ca/dgv/app/home), DECIPHER database (https://decipher.sanger.ac.uk/), and OMIM database (http://www.ncbi.nlm.nih.gov/omim). The found variants were further verified by Sanger sequencing.

## Results

### Intrauterine phenotypes

**Fig. 1:**
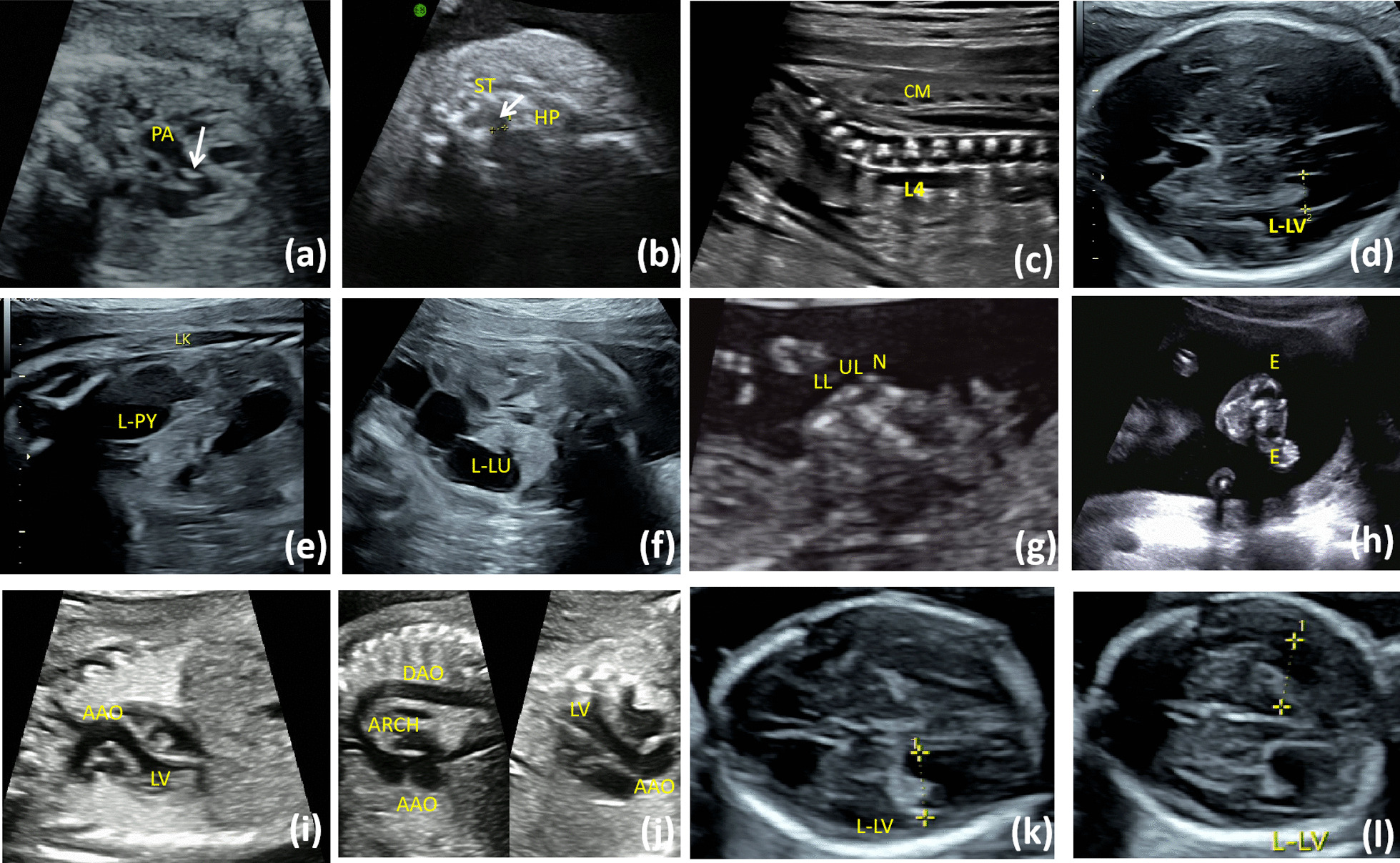
(**a**) The ultrasound examination of the first case with a hyperechogenic of fetal aortic valve (white arrow). **b** The ultrasound images of the second case in oblique view through oral fissure, showing break in echo of palatine bone horizontal plate(white arrow). **c** The ultrasound images of the second case showed a low-lying conus medullaris, the CM located at the L4 level. **d**, **k**, **l** The ultrasound view showed left mild ventriculomegaly in case 3(d), and bilateral mild ventriculomegaly in case 6 and 7(k,l). **e**, **f** The ultrasonic examination of the third case showed severe hydronephrosis of the left kidney with dilatation of the left ureter. **g**, **h** The ultrasonic examination of the fourth case showed an absent upper cranial vault and no cerebral tissue above the level of the orbits in the coronal view (**g**) and the coronal view (**h**). **i**, **j** The ultrasound images of the sixth and seventh cases showed the inner diameter of the ascending aorta was widened in the left ventricular outflow tract view and the long axis of the aortic arch view

A total of seven fetuses were diagnosed with 7q11.23 microduplication syndrome and their intrauterine phenotypes were assessed retrospectively. Clinical characteristics and genetic results are shown in Table 1. The following abnormalities were identified by prenatal ultrasonography: ventriculomegaly (Case 3, 6, 7 shown in Fig. 1d, k, l), low-lying conus medullaris (Case 2, 6, 7 shown in Fig. 1c), cleft palate (Case 2 shown in Fig. 1b), Dilated ascending aorta (Case 6, 7 shown in Fig. 1i, j), hyperechogenic aortic valve (Case 1 shown in Fig. 1a), anencephalus (Case 5 shown in Fig. 1g), severe hydronephrosis of the left kidney with dilatation of the left ureter (Case 3 shown in Fig. 1e, f), short long bones (Case 2). All the nuchal translucency thickness of the seven fetuses were < 95th percentile for their gestational age.

**Table 1:** Intrauterine phenotype features of fetuses with 7q11.23 microduplication syndrome

NO	Age	Intrauterine phenotypes	Prenatal diagnosis	Karyotype	CMA result		Inheritance	Trio-MES	Pregnancy outcome
					CNVs type	Sizes			
1	27	Hyperechogenic of fetal aortic valve	Amniocentesis for singleton	46, XX	arr[GRCh37] 7q11.23(72,600,482–74,175,485) × 3	1.5 Mb	De novo	–	TOP
2	28	Cleft palate, low-lying conus medullaris, shortened fetal long bones	Amniocentesis for singleton	46, XY	arr[GRCh37] 7q11.23(72,701,098–74,133,586) × 3	1.4 Mb	De novo	de novo heterozygous variants in COL2A1(NM_001844),c.3472G > A(p.G1158S)	TOP
3	23	Left ventriculomegaly, severe hydronephrosis of the left kidney with dilatation of the left ureter	Amniocentesis for singleton	46, XX	arr[GRCh37] 7q11.23(72752339_74146927) × 3	1.4 Mb	Unknown	–	Premature delivery by cesarean section. Birth weight was 2.1 kg
4	26	Anencephalus	Amniocentesis for MCDA twins	46, XY	arr[GRCh37] 7q11.23(72,701,098–74,069,645) × 3	1.4 Mb	De novo	–	TOP
5	26	–	Amniocentesis for MCDA twins	46, XY	arr[GRCh37] 7q11.23(72,701,098–74,069,645) × 3	1.4 Mb	De novo	–	
6	32	Bilateral ventriculomegaly, low-lying conus medullaris, dilated ascending aorta	Amniocentesis for MCDA twins	46, XY	arr[GRCh37] 7q11.23(72692113_74162823) × 3 16p13.11(14892976_16309046) × 3	1.5 Mb	Maternal	–	Premature delivery by cesarean section, their birth weight was 1.15 kg/1.25 kg, respectively
7	32	Bilateral ventriculomegaly, low-lying conus medullaris, dilated ascending aorta	Amniocentesis for MCDA twins	46, XY	arr[GRCh37] 7q11.23(72692113_74162823) × 3 16p13.11(14892976_16309046) × 3	1.5 Mb	Maternal	–	

TOP, termination of pregnancy

### Cytogenetic and molecular analyses

**Fig. 2:**
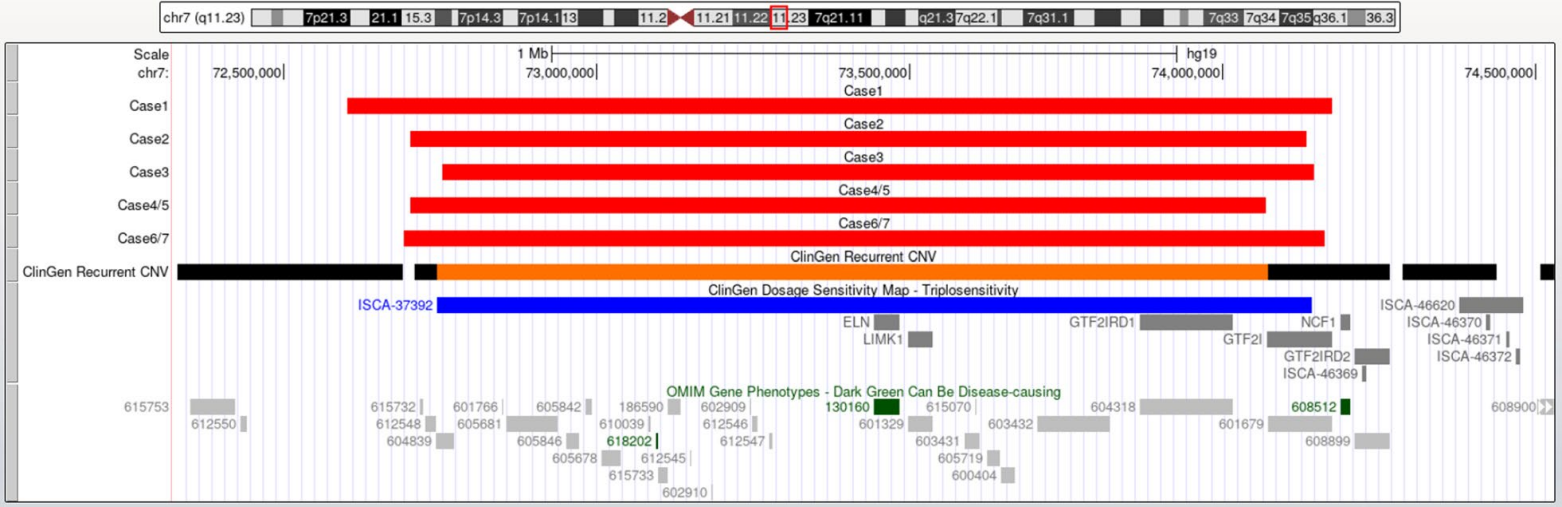
The breakpoints and covered genes of each microduplication of the seven fetuses, including the well-known OMIM disease-causing gene *ELN* (130,160) and NCF1 (608,512). All the cases consisted of typical deletions (1.40–1.55 Mb)

All 7 fetuses underwent G-banded karyotype analysis, no abnormal karyotype was found. They had different sizes and loci of chromosome microduplications in the 7q11.23 region, ranging from 1.4 to 1.5 Mb. The *ELN* gene duplications were identified by CMA in all 7 fetuses. The duplications in chromosome 7q11.23 are shown in Fig. 2. No other pathogenic microdeletions or microduplications were found. All the STR loci of the two pairs of MCDA twins were identical. The family of Case 2 accepted Trio-MES, and additionally detected a de novo heterozygous pathogenic variant c.3472G > A (p.G1158S) in *COL2A1* gene (NM_001844). Three pairs of parents (Case 1, 4–7) underwent CMA, and parents of Case 2 accepted Trio-MES. The results indicated that the dup7q11.23 of Case 6 and 7 were inherited from their asymptomatic mother, while in the other cases duplication was de novo.

### Follow ups

After genetic counseling, three pairs of parents (Case 1, 2, 4 and 5) requested to terminate the pregnancy. Parents of the other cases decided to continue the pregnancy. The infants were born prematurely by cesarean section (CS) for suspected fetal distress. Case 6 and 7 were delivered at 32^+5^ gestational age, their birth weights were 1.15 kg (2nd percentile)/1.25 kg (4th percentile), their head sizes were 29 cm (25th percentile)/29 cm (25th percentile), and their lengths were 39 cm (6th percentile)/40 cm (12th percentile). The appearances of the twin brothers are consistent with recognizable facial phenotypes, including straight and neatly placed eyebrows, broad forehead, and high broad nose. Their apgar score were 7 at 1 min after birth. After positive pressure ventilation, blood oxygen saturation was 80–82%, and the ph of umbilical cord blood indicated 7.18 and 7.21, respectively. Their apgar scores were both 9 and 9 at 5 and 10 min, so they were transferred to Neonatal Intensive Care Unit (NICU). Both twins showed mild ventriculomegaly on central nervous system MRI without epilepsy or vomiting. After symptomatic treatment, they were discharged after 21 days and 24 days, respectively. Regular check-ups showed dilation of the ascending aorta, but no other cardiac symptoms for the time being. Their growth and development were normal at one year of age. Fetus 3 was delivered at 32^+3^ gestational age. The newborn also had aberrant facieses, including broad nasal bridge, eyelid edema, and short philtrum. Her birth weight was 2.1 kg (72th percentile), head size was 31 cm (80th percentile), length was 44 cm (71st percentile). The APGAR scores of her was 9, 10 and 10 at 1, 5 and 10 min, respectively. The ph of umbilical cord blood indicated 7.3. She was transferred to NICU for premature birth, and was diagnosed with patent ductus arteriosus (PDA) and hydronephrosis. After symptomatic treatment, she was discharged after 7 days. She underwent laparoscopic pyeloplasty and transcatheter occlusion of patent ductus arteriosus at the age of 3 and 6 months, respectively. Postoperative follow-up ultrasound was performed every month. The cardiac ultrasound showed no obvious abnormality, but the urological ultrasound indicated the left kidney atrophy by degrees. Despite this, she had normal growth and development at one year old.

## Discussion

The chromosome 7q11.23 region is widely recognized because it contains the critical genes leading to Williams Beuren microdeletion and microduplication syndromes [10, 11]. It is flanked by 3 main clusters of low-copy repeats (LCR) named proximal (LCR-P), central (LCR-C), and distal (LCR-D), and it encompasses many and highly transcribed genes. Indeed, its gene density and transcriptional rate are significantly higher when compared with other segments of chromosome 7 [12]. To date, the genes between LCR-P and LCR-C have been marked as causative of these two disorders. The region involved in rearrangement is 1.4–1.5 Mb in length, encompassing about 26 to 28 genes, between the *NSUN5* gene on the centromeric end and *GTF2IRD2* gene on the telomeric end [10, 13, 14]. Notable genes include *ELN*, coding for elastic fibers, in connection with connective tissue abnormalities and cardiovascular disease; and *NCF1*, in connection with the risk of hypertension. Less than 100 cases of 7q11.23 microduplication syndrome have been described in the literature. Although the clinical phenotype of individuals with dup7q11.23 syndrome is not completely delineated, it is agreed that it is associated with milder and less distinct clinical features than its reciprocal microdeletion syndrome .[10, 13, 14]. Common clinical features of 7q11.23 duplication include delayed speech and developmental delay, learning difficulties, variable cognitive functions (from normal to intellectual disability), autism, characteristic facial features (broad forehead, high, broad nose, short philtrum, thin lips), growth issues (head size, height), congenital malformations (heart defects—PDA, subaortic stenosis, and aortic dilatation, renal anomalies, undescended testis, brain malformations), and epilepsy [3, 10, 15–18].

Most dup7q11.23 syndrome cases are diagnosed in childhood or adulthood due to language impairment, intellectual disability, mild facial dysmorphisms and congenital malformations [19]. However, very little prenatal information has been collected. A report by Dang et al. described two fetuses with dup7q11.23 syndrome carrying a typical 1.4–1.5 Mb duplication. One case with dup7q11.23 syndrome presented with lateral ventriculomegaly and polyhydramnios, while the other one showed choroid plexus cyst by prenatal ultrasonography [19]. Marcato et al. reported a fetus with dup7q11.23 syndrome, manifested with increased NT, absence of nasal bone, inversion of the “a” wave of the ductus venosus, and mild bilateral ventriculomegaly, lissencephaly-type abnormality [21]. Various congenital malformations were reported in the postnatal literatures, including many malformations which we assume that could be detected in prenatal ultrasonography, such as heart defects (subaortic stenosis and aortic dilatation), brain malformations (ventriculomegaly, agenesis of corpus callosum, posterior fossa cysts and cerebellar vermis hypoplasia), renal anomalies, cleft lip and/or palate [16–18]. Among fetuses diagnosed with 7q11.23 microduplication syndrome in our case series, the following intrauterine phenotypes were identified: ventriculomegaly (42.9%, 3/7), low-lying conus medullaris (42.9%, 3/7), dilated ascending aorta (28.6%, 2/7), cleft palate (14.3%, 1/7), hyperechogenic aortic valve (14.3%, 1/7), anencephalus (14.3%, 1/7), severe hydronephrosis of the left kidney with dilatation of the left ureter (14.3%, 1/7). In addition, fetal short long bones in Case 2 were suspected to be caused by the de novo pathogenic variant in *COL2A1*, which was associated with hypochondrogenesis type 2.

The structural malformations observed prenatally in our research were consistent with those reported in the literature. The ventriculomegaly was the most common sonographic feature observed in our case series, and was also reported in previous prenatal studies [20, 21]. The ventriculomegaly on brain imaging has been reported in several affected children and adult cases of dup7q11.23 syndrome as well [3, 16, 22, 23]. The ultrasound images of the fetus 2 showed a median cleft of the hard palate. It is a common congenital anomaly that has been reported in several affected children and adults [3, 17, 23]. Case 3 has severe hydronephrosis of the left kidney and dilatation of the ureter, which is consistent with two affected children in previous reports [10]. In our case series, there were three cases suspected of aortic disease. Case 1 had hyperechogenic aortic valve, Case 6 and 7 were MCDA twins with dilated ascending aorta (z-scores for the aorta were 1.95 and 2.09, respectively). Congenital cardiac defects have been reported in many pediatric and adult cases as well [16, 19], especially patent ductus arteriosus and aortopathy. Since patent ductus arteriosus is a postnatal diagnosis, it could not be identified prenatally. Parrott et al. presented a series of eight children and one adult with 7q11.23 microduplication syndrome, all of them had aortic dilation. The dilated ascending aorta was a rather specific abnormality repeatedly observed in pediatric and adult cases, and also in our prenatal cases. Moreover, three fetuses in our study showed a low-lying conus medullaris (CM). The CM position is important and abnormal position is associated with tethered cord, characterized by walking difficulty and sensory dysfunction in infants. Detection of a low-lying CM is essential in prenatal evaluation [24, 25], and we should stay alert if the CM below the L3 level after 23 weeks of gestation. In Fetus 2, the CM located at the L4 level at 24 weeks of gestation, but the fetus was aborted due to multiple malformations and no follow-up was available. Fetus 6 and 7 were MCDA twins both found low-lying CM at 24 weeks, the CM ascend to L2-3 at 32 weeks, and follow-up to 1 year old showed no associated symptom.

The *ELN* gene is considered the most critical and extensively explored gene in WBS. It encoded elastin protein (Elastin), which is a major component of the elastic fibers that strengthen connective tissue throughout the body. Its haplo-insufficiency is reported to cause supravalvular aortic stenosis (SVAS) in patients with WBS. The comparison have been made to the apparent opposite phenotypes caused by the 7q11.23 deletion and duplication, including opposing dysmorphic features and the relative strength/weakness in expressive language [17]. Moreover, SVAS was commonly observed in WBS, and aortic dilation was usually identified in individuals with 7q11.23 microduplication syndrome [17]. Here in our case series, this phenomenon was also found in prenatal cases (Case 6 and 7). While the SVAS in WBS has been attributed to ELN deletion, we postulate that a gene dosage effect of ELN may be the underlying cause for the prenatal dilated ascending aorta in these patients with the microduplication. However, the exact cause of aortic dilation in these patients remain unknown at this time.

Phenotype of 7q11.23 microduplication syndrome could be variable in the family series, ranging from apparent normal phenotype to delayed speech, autistic spectrum manifestations, intellectual disability, and cardiac abnormalities [26]. In the prenatal genetic counseling of 7q11.23 microduplication, it may not be accurate to predict the postnatal performance of offspring based on the symptoms of other positive members of the family. In our study, Case 6 and 7 were MCDA twins, and their 7q11.23 microduplication were inherited from their asymptomatic mother. They had typical facial features, dilation of the ascending aorta, and mild ventriculomegaly by the age of one year, follow-ups (cardiac, neural) on annual basis is onging. There was another pair of MCDA twins with 7q11.23 microduplication in our study, Case 4 and 5, but their prenatal progress were different: Case 4 diagnosed with anencephalus as early as 12 weeks of gestation, while Case 5 showed no apparent abnormality up to 20 weeks on labor induction. However, the prenatal phenotypes of case 6 and case 7 were consistent, including ventriculomegaly, low-lying CM, dilated ascending aorta at 24 weeks. Previous studies indicated that phenotypes of 7q11.23 duplication syndrome may vary significantly within a family, and this study further suggests that prenatal findings may be variable even for MCDA twins.

One limitation of our case series is that not all cases underwent exome sequencing to rule out additional molecular lesions. Case 2 is an example of how the short bones may have been erroneously reported as a potential association. We will conduct a thorough investigation to avoid potentially erroneous associations in our subsequent research.

## Conclusions

In summary, prenatal ultrasound findings of dup7q11.23 cases are diversified, with ventriculomegaly and low-lying conus medullaris being the most common intrauterine phenotypes. As frequently observed in adults and children cases, cleft palate, dilated ascending aorta, and renal abnormalities were also observed in the present prenatal cases, and should be pay attention to in subsequent studies. These findings may provide important clues for possible prenatal diagnosis of dup7q11.23 syndrome.

## Data Availability

The datasets generated and/or analyzed during the current study are not publicly available due individual privacy but are available from the corresponding author (Aihua Yin, E-mail: yinaihua0131@163.com) on reasonable request.

## References

[1] Morris CA, Thomas IT, Greenberg F (1993). Williams syndrome: autosomal dominant inheritance. Am J Med Genet.

[2] Sadler LS, Robinson LK, Verdaasdonk KR, Gingell R (1993). The Williams syndrome: evidence for possible autosomal dominant inheritance. Am J Med Genet.

[3] Berg JS, Brunetti-Pierri N, Peters SU, Kang SH, Fong CT, Salamone J (2007). Speech delay and autism spectrum behaviors are frequently associated with duplication of the 7q11.23 Williams-Beuren syndrome region. Genet Med.

[4] Mervis CB, Klein-Tasman BP, Huffman MJ, Velleman SL, Pitts CH, Henderson DR (2015). Children with 7q11.23 duplication syndrome: psychological characteristics. Am J Med Genet a.

[5] Abbas E, Cox DM, Smith T, Butler MG (2016). The 7q11.23 microduplication syndrome: a clinical report with review of literature. J Pediatr Genet.

[6] Merla G, Brunetti-Pierri N, Micale L, Fusco C (2010). Copy number variants at Williams-Beuren syndrome 7q11.23 region. Hum Genet.

[7] Stengel-Rutkowski S, Wirtz A, Hahn B, Hofmeister A, Murken JD (1976). Routine G-banding in prenatal diagnosis of chromosomal disorders. Hum Genet.

[8] Xiang J, Ding Y, Song X, Mao J, Liu M, Liu Y (2020). Clinical utility of SNP array analysis in prenatal diagnosis: a cohort study of 5000 pregnancies. Front Genet.

[9] Vetro A, Pisano T, Chiaro S, Procopio E, Guerra A, Parrini E (2020). Early infantile epileptic-dyskinetic encephalopathy due to biallelic PIGP mutations. Neurol Genet.

[10] Zarate YA, Lepard T, Sellars E, Kaylor JA, Alfaro MP, Sailey C (2014). Cardiovascular and genitourinary anomalies in patients with duplications within the Williams syndrome critical region: phenotypic expansion and review of the literature. Am J Med Genet a.

[11] Morris CA, Mervis CB, Paciorkowski AP, Abdul-Rahman O, Dugan SL, Rope AF (2015). 7q11.23 duplication syndrome: physical characteristics and natural history. Am J Med Genet a.

[12] Ebert G, Steininger A, Weissmann R, Boldt V, Lind-Thomsen A, Grune J (2014). Distribution of segmental duplications in the context of higher order chromatin organisation of human chromosome 7. BMC Genomics.

[13] Antonell A, Del CM, Magano LF, Kaufmann L, de la Iglesia JM, Gallastegui F, (2010). Partial 7q11.23 deletions further implicate GTF2I and GTF2IRD1 as the main genes responsible for the Williams-Beuren syndrome neurocognitive profile. J Med Genet.

[14] Faundes V, Santa ML, Morales P, Curotto B, Parraguez MM (2016). Distal 7q11.23 duplication, an emerging microduplication syndrome: a case report and further characterisation. Mol Syndromol.

[15] Torniero C, Dalla BB, Novara F, Cerini R, Bonaglia C, Pramparo T (2008). Dysmorphic features, simplified gyral pattern and 7q11.23 duplication reciprocal to the Williams-Beuren deletion. Eur J Hum Genet.

[16] Van der Aa N, Rooms L, Vandeweyer G, van den Ende J, Reyniers E, Fichera M (2009). Fourteen new cases contribute to the characterization of the 7q11.23 microduplication syndrome. Eur J Med Genet.

[17] Dixit A, McKee S, Mansour S, Mehta SG, Tanteles GA, Anastasiadou V (2013). 7q11.23 Microduplication: a recognizable phenotype. Clin Genet.

[18] Parrott A, James J, Goldenberg P, Hinton RB, Miller E, Shikany A (2015). Aortopathy in the 7q11.23 microduplication syndrome. Am J Med Genet a.

[19] Dentici ML, Bergonzini P, Scibelli F, Caciolo C, De Rose P, Cumbo F *et al*. 7q11.23 microduplication syndrome: clinical and neurobehavioral profiling. Brain Sci 2020, 10(11). 10.3390/brainsci10110839 PMC7697259 33187326

[20] Dang Y, Wan S, Zheng Y, Song T, Li C, Li Y (2020). The prenatal diagnosis of seven fetuses with 7q11.23 microdeletion or microduplication. Fetal Pediatr Pathol.

[21] Marcato L, Turolla L, Pompilii E, Dupont C, Gruchy N, De Toffol S (2014). Prenatal phenotype of Williams-Beuren syndrome and of the reciprocal duplication syndrome. Clin Case Rep.

[22] Depienne C, Heron D, Betancur C, Benyahia B, Trouillard O, Bouteiller D *et al*. Autism, language delay and mental retardation in a patient with 7q11 duplication. BMJ Case Rep 2009, 2009. 10.1136/bcr.05.2009.1911 PMC3028180 21686962

[23] Orellana C, Bernabeu J, Monfort S, Rosello M, Oltra S, Ferrer I *et al*. Duplication of the Williams-Beuren critical region: case report and further delineation of the phenotypic spectrum. BMJ Case Rep 2009, 2009. 10.1136/bcr.06.2009.1996 PMC3027765 21731584

[24] Zhai J, Cai A, Wei Q, Xie L, Jing C (2019). A method for quantitative 2-dimensional sonographic analysis of the fetal conus medullaris position. J Ultrasound Med.

[25] Lam WW, Ai V, Wong V, Lui WM, Chan FL, Leong L (2004). Ultrasound measurement of lumbosacral spine in children. Pediatr Neurol.

[26] Patil SJ, Salian S, Bhat V, Girisha KM, Shrivastava Y, Vs K (2015). Familial 7q11.23 duplication with variable phenotype. Am J Med Genet A.

